# Factors associated with the prevalence of back pain and work absence in shipyard workers

**DOI:** 10.1186/s12891-018-1931-z

**Published:** 2018-01-11

**Authors:** Seiji Watanabe, Toshiaki Takahashi, Jun Takeba, Hiromasa Miura

**Affiliations:** 1 Department of Orthopaedic Surgery, Ehime University Graduate School of Medicine, Toon, Ehime 791-0295 Japan; 2 Community Medical Support Center, Ehime University Graduate School of Medicine, Toon, Ehime 791-0295 Japan

**Keywords:** Back pain, Risk factor, Shipyard workers, Working environment, Questionnaire, Absence

## Abstract

**Background:**

We conducted a questionnaire survey of shipyard workers to identify difficulties experienced due to orthopedic or musculoskeletal disorders.

**Methods:**

The subjects were 375 workers (male, 361; female, 14) who worked for a single shipbuilding company. Questionnaire items covered the working environment, including work environment, working posture, and the weight of objects that the subject dealt with, as well as physical and lifestyle characteristics, namely smoking habits, drinking habits, sleeping hours, medications, exercise habits, and any weight gain of 20 kg or more since the age of 20. Subjects were also asked to indicate if they regularly experienced any of 17 listed difficulties in their daily lives, and to use an illustration of the human body to mark any body parts that were painful or hard to move.

**Results:**

The mean age was 41.8 years (19–73 years). The lower and/or upper back was the most frequent site of pain (46.5%), followed by the shoulders (11.4%), knees (9.6%), and neck (5.3%). Maintaining a half-sitting posture was the most problematic activity of daily living. Back pain was less frequent in subjects who exercised regularly, and more common in those who worked with heavy loads or in narrow spaces. A multinomial logistic regression analysis showed that absence from work was more common in subjects with back pain who had gained weight since their youth, who smoked, who used fire while welding metal, or who worked in a lying posture. While 35.4% of subjects had experienced absence from work due to musculoskeletal pain, only 5.1% were permitted by their employer to alter their work content or reduce their workload.

**Conclusions:**

These results indicate that a large number of shipyard workers have difficulties in their work and daily life activities due to back pain. To prevent worsening of pain and to reduce work absence, it is important to provide appropriate training to minimize the risk factors for back pain that were identified in this study.

## Background

Workers involved in manufacturing often labor in physically harsh environments. Low back pain arising from ergonomic exposures at work is an important cause of disability [1]. Of all 291 conditions studied in the Global Burden of Disease 2010 Study, low back pain ranked highest in terms of causing disability [2]. Musculoskeletal disorders due to continuous load bearing cause work absences that decrease the productivity not only of workers but also of companies [3, 4]. It was previously reported that musculoskeletal disorders caused high absence rates in company employees [5]. Thus, it is important to identify risk factors for these disorders in the areas of lifestyle and the work environment, and to educate workers about these factors to reduce their vulnerability.

Workers experience various musculoskeletal difficulties related to occupational and psychosocial factors [6, 7]. Andersen et al. reported that musculoskeletal pain, especially hand or wrist pain and low back pain, was a risk factor for long-term work absence. They also pointed out that neck and shoulder pain was common in white collar workers [8, 9]. In particular, the prevalence of low back pain was found to be relatively high among shipyard workers [10], and was influenced by psychological and psychosocial factors [11, 12]. Low back pain causes both absence from work [13] and low productivity. Therefore, an appropriate approach to musculoskeletal disorders such as low back pain is critically important [14–16].

Several disorders have been reported among shipyard workers [17–19]. In particular, the prevalence rate of musculoskeletal disorders in shipyard workers is estimated to be high because they must often adopt awkward postures and lift heavy loads. However, very few studies have investigated this population’s experience with musculoskeletal disorders, namely injuries or pain in the bones and joints, and related medical consultations [20, 21].

The purpose of this study was first to conduct a questionnaire survey of shipyard workers, and based on the results, to evaluate the factors associated with the prevalence of back pain and work absence due to back pain in shipyard workers.

This study obtained data on (1) each subject’s lifestyle and exercise habits, work environment, medical history, history of absence from work, and sites of musculoskeletal disorders and resulting difficulties; and (2) the relationship between back pain and subjects’ lifestyle and exercise habits.

## Methods

### Subjects

A total of 436 employees (male, 417; female, 19) at a single shipbuilding company were solicited to participate in this study. Of these, 375 (male, 361; female, 14) consented to complete the questionnaire survey; their mean age was 41.8 years (19–73 years). We received valid responses from all 375 workers. This was a cross-sectional study, and the questionnaire was developed and designed by a study author (TT).

### Assessment

Our questionnaire covered the following items, which are shown in Table 1: the physical characteristics and lifestyle category included smoking habits, drinking habits, sleeping hours, medications, exercise habits, and any weight gain of 20 kg or more since the age of 20; the working environment category included work environment, working posture, and the weight of objects that the subject had to lift on a regular basis. The questions on work environment and working posture allowed subjects to provide multiple answers.

**Table 1:** Questionnaire items related to the work environment

The environment of the workplace: (1) Often work outdoors (2) The floor of the workplace is unstable (e.g., slippery or rugged) (3) Often exposed to significant trembling, vibration, or impact (4) Often work in a noisy environment (5) Often work in a narrow space (6) Use fire during work
Working posture: (1) Often work in a seated posture (2) Often work in sitting posture (3) Often work in a half-sitting posture (4) Often work in a standing posture (5) Often twist the waist during work (6) Often bend forward at the waist to an extreme degree (7) Often raise hands above shoulders or extend arms fully (8) Sometimes work in a lying posture
The weight of carried loads (per person): (1) Mainly 40 kg or more (2) Mainly 20–40 kg (3) Mainly 10–20 kg (4) Do not handle heavy loads

This study focused on the prevalence of back pain, as assessed with the following question: “Has back pain ever interfered with your work?” We divided the responses into four groups: “no,” “only in the past,” “sometimes (currently),” and “always.” Back pain was considered to be present if the subject answered “sometimes (currently),” or “always.”

Subjects were also asked 17 questions (shown in Table 2) about difficulties experienced in their daily lives, and instructed to place check marks next to any applicable items. They were also asked to indicate any body parts that were painful or that they had difficulty moving by drawing an “X” on a schema (an illustration of the human body). Based on the participants’ responses on the schema, we classified body regions into the neck, shoulder, elbow, upper arm, forearm, wrist joint, hand, back, lower back, hip joint, thigh, knee, lower leg, foot, and ankle joint.

**Table 2:** Survey items on activities of daily living

(1) Fixing hair
(2) Tying knots behind the back
(3) Opening and closing a sliding door
(4) Putting hand to mouth
(5) Raising hands above shoulders
(6) Grasping something with fingers
(7) Working with the neck bent
(8) Prolonged work in a standing posture
(9) Remaining in a half-sitting posture
(10) Repeatedly squatting and standing up
(11) Turning over while sleeping
(12) Standing up
(13) Washing face
(14) Maintaining a sitting posture for a long period (one hour or longer)
(15) Lifting a heavy load
(16) Walking 1 km or longer
(17) Going up or down stairs

### Statistical analysis

This study focused on back pain. We performed binomial and multinomial logistic regression analysis with SPSS for Windows (Statistical Package for Social Science, IBM Inc.) to examine the relationship between the existence of pain and the following factors: the subject’s lifestyle habits, work environment, working posture, history of medical or alternative medical consultations and therapy, work absence, and changes in the work environment. We also examined the relationship between the existence of back pain and work absence. Differences were considered significant for *P* < 0.05.

## Results

The mean height of participants was 168.0 ± 6.6 cm, and the mean weight was 67.2 ± 12.3 kg. In terms of lifestyle category data, 54.5% of the subjects had a smoking habit: 4.0% smoked occasionally, 35.9% smoked 20 or fewer cigarettes a day, and 14.6% smoked more; 45.5% did not smoke at all. The mean sleep duration per night was 6 h and 50 min prior to workdays, and 7 h and 30 min before days off. Regarding quality of sleep, 23.9% rated it as “good,” 62.0% as “ordinary,” and 5.3% as “bad.”

Twelve percent of the subjects had gained 20 kg or more since the age of 20, and 20.5% took medications regularly. Furthermore, 57.4% did not exercise regularly, while 11.2% exercised once to twice per month, 19.4% exercised one to three times per week, and 7.2% exercised four or more times per week. Finally, 14.1% of subjects had maintained a vigorous exercise routine (involving continuous sweating) for 1 year or longer, for 30 min or more at least two times per week.

In terms of weight carried, 35.6% of the subjects handled almost no heavy loads, while more than 55% lifted loads of 10 kg or more: 40.4% lifted mainly 10–20 kg, 10.1% lifted mainly 20–40 kg, and 4.3% lifted mainly 40 kg or more.

Subjects had difficulty with the following activities of daily living (ADLs): keeping a half-sitting posture (37.2%), maintaining a sitting position for long periods (1 hour or longer) (23.1%), lifting heavy loads (17.3%), squatting and standing repeatedly (16.0%), working in a standing posture for long periods (12.0%), going up or down stairs (10.1%), working with the neck bent (9.0%), standing up (5.9%), walking 1 km or longer (5.1%), tying knots behind their back (3.2%), washing their face (2.9%), raising hands above the shoulders (2.7%), turning over while sleeping (2.4%), grasping something with the fingers (1.6%), fixing their hair (1.1%), opening and closing a sliding door (0.8%), and putting their hands to their mouth (0.3%).

Pain in the back interfered with work in 46.5% of subjects (“occasionally” in 40.4% and “continuously” in 6.1%). Other body parts that were painful or hard to move, as indicated using the schema, included the following: shoulder (11.4%), knee (9.6%), neck (5.3%), elbow (3.4%), foot and ankle joint (2.6%), hand or wrist joint (2.5%), upper arm or forearm (0.9%), hip joint (0.5%), lower leg (0.3%), and thigh (0.3%).

Of the 46.5% of subjects in whom back pain interfered with work either “sometimes (currently),” or “always,” 36.4% had consulted a medical professional due to the pain; in addition, 41.5% had received alternative medical treatment (e.g., acupuncture, moxibustion, massage, and bone setting). Also, 35.4% of the subjects had experienced absence from work due to back pain, but in only 5.1% of cases did their company change their work content or reduce their workload.

We analyzed the relationship between the existence of back pain and lifestyle habits using the odds ratio (OR). The prevalence rate of back pain in subjects who engaged in vigorous exercise (30 min or longer, at least twice per week) was low (OR = 0.602). On the other hand, the OR for the weight of the load carried at work was 2.817, identifying this as a risk factor for pain **(**Table 3). Statistically significant lifestyle-related risk factors for work absence due to back pain were an increase in smoking (OR = 1.616), weight of carried loads (OR = 1.854), and body weight gain (20 kg or more since the age of 20) (OR =1.942) **(**Table 3).

**Table 3:** Factors associated with the prevalence of back pain and work absence in shipyard workers-lifestyle-related factors

	Existence of pain *n* = 175		Absence from work *n* = 132	
	OR ICC95%	*P* value	OR ICC95%	*P* value
・Smoking	1.436 0.953–2.163	0.084	1.616 1.046–2.497	0.030
・Hous of sleep (work day)	1.063 0.876–1.289	0.537	1.033 0.862–1.238	0.724
・Hous of sleep (holiday)	1.099 0.941–1.284	0.232	0.944 0.746–1.195	0.632
・Exercise habit	0.877 0.698–1.101	0.258	1.105 0.715–1.708	0.654
・Weight of carried loads	2.817 1.845–4.302	0.000	1.854 1.199–2.867	0.006
・Body weight gain(20 kg or more since age 20)	1.492 0.794–2.803	0.214	1.942 1.031–3.656	0.041
・Vigorous exercise(with continuous sweating)^a^	0.602 0.329–1.105	0.101	0.965 0.519–1.795	0.910

^a^for 30 minuites or more per day, twice a week, for one year

*OR* odds ratio

Regarding the relationship between back pain and working environment, working in narrow spaces and using fire during work were identified as significant positive risk factors (OR = 1.912 and 1.637, respectively). Use of fire was the one risk factor related to the workplace environment that was significantly correlated with work absence due to back pain (OR = 2.116) **(**Table 4). There was a significant relationship between back pain and frequently working in a half-sitting posture (OR = 2.241) or bending forward at the waist to an extreme degree (OR = 2.271). Sometimes working in a lying posture was a risk factor for absence from work due to back pain (OR = 2.420) **(**Table 5).

**Table 4:** Factors associated with the prevalence of back pain and work absence in shipyard workers-working environment

		Existence of pain		Absence from work	
		*n* = 175		*n* = 132	
	Frequency (%)	OR ICC95%	*P* value	OR ICC95%	*P* value
・Often work outdoors	27.4	1.118 0.692–1.807	0.648	1.567 0.963–2.549	0.070
・Work on unstable floor	31.4	1.131 0.677–1.890	0.639	0.998 0.586–1.700	0.995
・Expose to significant trembling, vibration, or impact	17.6	1.115 0.629–1.974	0.710	0.595 0.322–1.098	0.097
・Often work in a noisy environment	60.1	1.252 0.776–2.019	0.357	0.840 0.506–1.393	0.499
・Work in narrow space	18.9	1.912 1.094–3.343	0.023	1.359 0.775–2.381	0.284
・Use fire at work	43.6	1.637 1.017–2.634	0.042	2.116 1.285–3.485	0.003

*OR* odds ratio

**Table 5:** Factors associated with the prevalence of back pain and work absence in shipyard workers-working posture

		Existence of pain *n* = 175		Absence from work *n* = 132	
	Frequency (%)	OR ICC95%	*P* value	OR ICC95%	*P* value
・Often work in a seated posture	15.4	0.661 0.343–1.274	0.216	0.573 0.291–1.126	0.106
・Often work in a sitting posture	25.5	1.271 0.760–2.126	0.360	0.956 0.569–1.608	0.865
・Often work in a half-sitting posture	39.9	2.241 1.363–3.684	0.001	1.302 0.782–2.166	0.311
・Often work in a standing posture	42.3	0.922 0.572–1.486	0.739	0.682 0.419–1.108	0.112
・Often twist the wait during work	14.9	0.929 0.454–1.903	0.841	1.412 0.697–2.858	0.338
・Often bend forward at the waist an extreme degree	10.4	1.516 0.665–3.358	0.322	1.109 0.498–2.467	0.800
・Often raise hands above the shoulders or extend arms	17.3	2.271 1.111–4.641	0.024	0.745 0.364–1.525	0.420
・Sometimes work in a lying posture	14.4%	0.922 0.461–1.844	0.818	2.420 1.223–4.789	0.011

*OR* odds ratio

As shown in Table 6, back pain and absence from work were associated with other factors such as medical or alternative medical consultations and change in work content/workload. The OR for consulting a medical professional was 2.126 and that for seeing an alternative medical provider was 3.523 (Table 6). The OR for missing work due to back pain in subjects who consulted a physician was very high at 11.796, compared to 2.211 in those who consulted with an alternative medical provider. The OR for changing work content or reducing workload was 6.528 (Table 6).

**Table 6:** Factors associated with the prevalence of back pain and work absence in shipyard workers-other factors

		Existence of pain *n* = 175		Absence from work *n* = 132	
	Frequency	OR ICC95%	*P* value	OR ICC95%	*P* value
・Consulted a medical doctor	36.4%	2.126 1.142–3.956	0.017	11.796 6.412–21.703	0.000
・Consulted an alternative medical provider ^a^	41.5%	3.523 2.067–6.006	0.000	2.211 1.181–4.141	0.013
・Occurrence of absence from work	35.4%	1.802 0.978–3.321	0.059	–	–
・Change in work content or reduction workload	5.16%	4.305 0.926–20.014	0.063	6.528 1.503–28.343	0.012

^a^acupuncture, moxibustion, massage, and bone setting

## Discussion

IJzelenberg et al. [22] identified risk factors for musculoskeletal symptoms associated with work, taking into account the relationship between symptoms and workers’ histories of medical consultation as well as the occurrence of work absence. They reported that the risk factors for low back pain were old age and living alone, and those for neck and upper extremity pain were living alone and female gender. Analyzing the reasons for workers’ musculoskeletal pain and then eliminating these causes or reducing their impact should contribute to the increased productivity of society overall [3, 4].

We thus considered the prevalence of musculoskeletal disorders among shipyard workers, most of whom are manual laborers. We found that back pain was the most common condition in these workers and that the pain had interfered with work. We then examined the relationship between back pain and the workers’ daily habits and work environments.

Regarding the relationship between back pain and work absence, this study identified smoking, weight gain (20 kg or more since age 20), and the weight of carried loads as significant risk factors for work absence. Several previous studies also reported a causal relationship between smoking and lower back pain [22–26]. About half of the subjects in our study smoked, and we thus consider that smoking cessation assistance should be provided so as to improve future life habits. Also, Oleske et al. [23] reported that smoking worsened low back pain in automotive industry workers. In agreement with our results, a previous study reported that body weight gain was a risk factor for low back pain [27]. Heuch et al. [28] identified a significant, positive causal relationship between low back pain and obesity. Based on these results, it is important to provide individuals with musculoskeletal disorders such as chronic low back pain with instructions on smoking cessation and weight control [29, 30]. The weight of carried loads was correlated with both the presence of back pain and absence from work, whereas body weight gain was not correlated with the existence of back pain, but absence from work.

Miranda et al. [31] reported that among workers younger than 50, low back pain was related to physical workload (a composite measure that included heavy lifting, awkward postures, and whole-body vibration), while in workers aged 50 and older, this pain was correlated with health behavior (the sum of smoking, excessive body weight, and lack of physical exercise).

In our study, the most common ADL-related difficulty was maintaining a half-sitting posture, followed by remaining in a sitting posture for a long period of time (1 hour or more), lifting heavy loads, and squatting or standing repeatedly, all of which are highly necessary during work in shipyards. Thus, it was assumed that work-related burdens interfered with our subjects’ endeavors in everyday life.

Pain was most commonly experienced in the back, followed by the shoulders and neck, which was consistent with earlier findings [20]. Several previous studies [17–19, 21] administered questionnaires or conducted medical interviews in cohorts comprised of shipyard workers. Alexopoulos et al. [32] found that work absence due to low back pain was most likely in individuals who were unmarried, worked night shifts, and had low levels of education. In contrast, the results of this study identified smoking, weights of carried loads, and body weight gain as risk factors. These discrepancies are due to variations in questionnaire items, and we should evaluate the items in their study in the future.

The work environments identified in this survey were harsh (often noisy, involving fire, etc.), and subjects had to adopt a variety of work postures that included not only standing and half-sitting postures, but also lying supine or prone (14%). Over half the subjects lifted loads of 10 kg or more (per person), which are considered significant from a physiological point of view. In this study, work in narrow spaces and use of fire (while welding metal) were correlated with the presence of back pain, but only use of fire was correlated with absence from work (Table 4). Work in narrow spaces requires that workers adopt awkward postures, and thus our findings agree with those of the abovementioned report by Miranda et al. [31]. The use of fire, which is common in shipyards, stresses workers’ backs because it necessitates maintaining a tense and motionless squatting posture.

Lund et al. [24] reported that uncomfortable work postures (extreme bending, twisting of the neck or back, working mainly while standing or squatting, etc.) contributed to prolonged work absences. In our survey, back pain was significantly related to frequently working in a half-sitting posture and to commonly raising the hands above the shoulders or extending the arms (Table 5). Working in a lying posture was identified as a risk factor for absence from work (Table 5). We assumed that this factor caused pain by similar mechanisms as working in narrow spaces.

Harkness et al. [25] identified the following work postures as significant risk factors for low back pain in newly employed workers: lifting heavy loads, pulling objects, kneeling, and squatting. Matsudaira et al. [26] studied the incidence of low back pain over a period of 2 years and found that a history of low back pain, repeated lifting actions, and repetitive work were all significant risk factors. Our study found that back pain was made worse by heavy carried loads (Table 3), the use of fire (Table 4), and working in a half-sitting position (Table 5).

Our survey showed that a large proportion of workers with back pain had received medical or alternative medical consultations. The OR for absence from work was 11.8 in those who consulted a medical doctor, and 2.2 in those who consulted an alternative medical provider. Among all the risk factors studied, pain-related visits to a physician correlated most strongly with absence from work. We assume this was due to the fact that those who requested medical consultations had more severe pain than those who visited alternative medical providers.

Fortunately, preventive and treatment measures for back pain are often effective. Andersen et al. [9] studied workers with musculoskeletal disorders and found that while 92% reported symptoms, most had a good prognosis. Oleske et al. [23] reported that low back pain in automotive industry workers was relieved by stress reduction, exercise, and physical activities other than work, and thus they encouraged these workers to exercise and reduce stress to lessen low back pain. Regularly changing positions at work has been recommended for reducing low back pain because work environment and psychosocial factors are related to the progression of symptoms [33]. Mental stress has also been found to cause or worsen low back pain [34], and thus providing mental health care in the workplace should be considered.

Our survey indicated that 5.1% of workers with back pain were successful in getting their companies to change their work content or reduce their workload. Although these changes may be difficult in practice, depending on the severity of pain they may be more effective if implemented promptly and in consultation with workers [35]. Even in cases where workers have severe pain but have not yet been absent from work, changing the work content or environment should be recommended to prevent any future absences [36].

### Limitations

This study used a cross-sectional design, which has limitations in reliability and repeatability because questionnaire data are based on participants’ memories. The 17 items in the questionnaire were developed by the authors, and their validity and reliability remain to be examined. Back pain is a subjective complaint; several reports have pointed out the importance of psychological and psychosocial factors in musculoskeletal disorders, and these were not evaluated in this questionnaire survey.

To help prevent musculoskeletal disorders in the future, it is important that questionnaire and medical interview results be compared with those of direct medical examinations to ensure adequate consistency. We recommend that employers initiate workplace health promotion interventions and also provide advice for employees.

## Conclusions

This study found that back pain was the most common musculoskeletal disorder among shipyard workers, and it resulted in an absence from work in 35% of participants. It was also found that increased body weight, current smoking, lifting loads, and working in a half-sitting posture were major risk factors for back pain. It is considered important to take earlier measures to avoid absence from work due to back pain.
